# Relationship of aerobic fitness and motor skills with memory and attention in preschoolers (Ballabeina): A cross-sectional and longitudinal study

**DOI:** 10.1186/1471-2431-11-34

**Published:** 2011-05-11

**Authors:** Iris Niederer, Susi Kriemler, Janine Gut, Tim Hartmann, Christian Schindler, Jérôme Barral, Jardena J Puder

**Affiliations:** 1 Institute of Exercise and Health Sciences, University of Basel, Birsstrasse 320b, 4052 Basel, Switzerland; 2 Swiss Tropical and Public Health Institute, University of Basel, Socinstrasse 57, 4002 Basel, Switzerland; 3 Institute of Psychology Basel, University of Basel, Socinstrasse 57, 4002 Basel, Switzerland; 4 Institute of Sport Sciences, University of Lausanne, Bâtiments administratifs de Vidy, Route de Chavannes 33, 1015 Lausanne, Switzerland; 5 Service of Endocrinology, Diabetes and Metabolism, University of Lausanne, Centre Hospitalier Universitaire Vaudois, Rue du Bugnon 46, 1011 Lausanne, Switzerland

## Abstract

**Background:**

The debate about a possible relationship between aerobic fitness and motor skills with cognitive development in children has recently re-emerged, because of the decrease in children's aerobic fitness and the concomitant pressure of schools to enhance cognitive performance. As the literature in young children is scarce, we examined the cross-sectional and longitudinal relationship of aerobic fitness and motor skills with spatial working memory and attention in preschool children.

**Methods:**

Data from 245 ethnically diverse preschool children (mean age: 5.2 (0.6) years, girls: 49.4%) analyzed at baseline and 9 months later. Assessments included aerobic fitness (20 m shuttle run) and motor skills with agility (obstacle course) and dynamic balance (balance beam). Cognitive parameters included spatial working memory (IDS) and attention (KHV-VK). All analyses were adjusted for age, sex, BMI, migration status, parental education, native language and linguistic region. Longitudinal analyses were additionally adjusted for the respective baseline value.

**Results:**

In the cross-sectional analysis, aerobic fitness was associated with better attention (*r *= 0.16, *p *= 0.03). A shorter time in the agility test was independently associated with a better performance both in working memory (*r *= -0.17, *p *= 0.01) and in attention (*r *= -0.20, *p *= 0.01). In the longitudinal analyses, baseline aerobic fitness was independently related to improvements in attention (*r *= 0.16, *p *= 0.03), while baseline dynamic balance was associated with improvements in working memory (*r *= 0.15, *p *= 0.04).

**Conclusions:**

In young children, higher baseline aerobic fitness and motor skills were related to a better spatial working memory and/or attention at baseline, and to some extent also to their future improvements over the following 9 months.

**Trial Registration:**

clinicaltrials.gov NCT00674544

## Background

The debate about a possible relationship between aerobic fitness and motor skills with cognitive development in children has recently re-emerged, because of the decrease in children's aerobic fitness [1] and the concomitant pressure of schools to enhance cognitive performance [2]. In preschoolers, the focus is preferentially set on the advancement of cognitive parameters such as working memory and attention. Both working memory and attention have been shown to be important predictors of academic achievement [3,4].

Despite the beneficial health effects, time for physical education or sports activities is often limited by budget cuts or the abundance of alternative out-of-school offers [5]. These trends may already affect the level of activity and subsequent aerobic fitness in young children.

It has been suggested that motor and cognitive development may be more interrelated than has previously been appreciated [6]. The cerebellum is not only important for motor but also for those cognitive functions, that are related to the dorsolateral prefrontal cortex [6]. Currently, there are three hypotheses based on data in rodents and humans explaining how exercise may affect cognitive parameters [7]: (1) increase in **oxygen saturation **based on an increased blood flow and angiogenesis, (2) increase in **brain neurotransmitters **like serotonin and norepinephrine facilitating information processing and (3) **regulation of neurotrophins **such as different growth factors. Additionally we know from studies in rodents, that exercise stimulates neurogenesis in the hippocampus and the subventricular zone [7] which may be important for lasting and cumulative network adaptations [8].

Studies in primate and adult humans have shown a relationship between aerobic fitness and cognitive performance [7]. These findings have been recently confirmed in a study including more than one million adolescents [9], where aerobic fitness was positively associated with intelligence at baseline and changes in aerobic fitness between age 15 and 18 years independently predicted intelligence at age 18 [9]. Similar cross-sectional relationships between aerobic fitness and measures of cognitive performance such as attention and working memory have been found in preadolescent schoolchildren [10-12], while there are no data in preschoolers. However, while aerobic fitness is considered to be the main parameter responsible for cognitive benefits in adults, data from recent cross-sectional studies in preadolescent children demonstrate that not only aerobic fitness, but also other domains like motor skills (e.g. balance, agility, ball skills) seem to be related to cognitive benefits in children [13-17]. Furthermore, exercises involving specific mental processing including executive functions might be more prone to trigger global cognitive development in children than aerobic exercises alone [18,19].

But research in children shows generally mixed results. While cross-sectional studies point towards a positive relation between aerobic fitness and/or motor skills with cognitive performance [12-18,20], longitudinal data [18,21] and intervention studies [18,20] are few and more inconclusive [18]. Tomporowski and colleagues assume different causes in the sometimes contradictory results of the studies in children [18]: Lack of sensitivity of the selected tests to evaluate motor and cognitive performance, substantial differences among populations in the different studies and effect dependency according to age and developmental level. They also postulate that specific types of exercise training may facilitate cognitive performance more than others. Other causes for the contradictory results might be that some of the studies were limited by self reported or subjectively assessed data [21], measures of aerobic fitness or motor skills often restricted to one domain [12,14,17], analyses not adjusted for age and sex [14] and that other confounder variables like BMI or socioeconomic status were only rarely taken into account [21,22]. Thus, more comprehensive cross-sectional and longitudinal analyses are needed. In preschoolers, studies are particularly rare [13,14,17] and controversial [17] and there is a lack of longitudinal data.

We therefore comprehensively assessed the cross-sectional and longitudinal relationships of aerobic fitness and motor skills (agility, dynamic balance) with two different cognitive parameters (spatial working memory and attention) in a sample of ethnically diverse Swiss preschool children controlling for sociocultural characteristics and for BMI. Based on the literature, our **hypothesis was that higher aerobic fitness and better motor skills in young children would be related to better memory and attention at baseline and to their improvements over 9 month. We also hypothesized that the relationship would vary according to the investigated domains**.

## Methods

We analyzed data from a randomized controlled trial (Ballabeina Study, clinicaltrials.gov NCT0067454; [23]), involving preschoolers from the German (St. Gallen) and French speaking part (Vaud) of Switzerland [23]. Children were assessed both at baseline (summer 2008) and 9 months later. Children from the 20 control classes were used for these analyses. They did not receive any intervention and were not at the same schools as the intervention classes. The study was approved by the cantonal ethical committees of St. Gallen and Vaud and written informed consent from the parents or legal representatives was obtained for 312 of the initial 367 control children (participation rate: 85%). The current database focuses on those 245 children (79% of the 312 participating children) with aerobic fitness and cognitive data at both time points. Compared to children whose parents consented but who did not have a complete valid dataset (*n = *67), the 245 children in the current analysis were 0.2 years older (*p *= 0.02) and more likely to have parents of low educational level (44 vs. 26%, *p *= 0.02). Otherwise they did not significantly differ regarding sex, BMI, baseline aerobic fitness, motor skills and cognitive and sociocultural characteristics. Complete information about sociocultural characteristics was available for 217 of 245 children so that the adjusted analyses were done with this reduced sample.

### Aerobic fitness and motor skills

The tests of aerobic fitness, agility and dynamic balance were assessed in a gym during a time period of 45-50 minutes. After a standardized warm up, children were divided in two groups: One performed first the obstacle course and then the balance beam test and vice versa. For both motor skill tests, children were divided in groups consisting each of three to four children headed by a trained researcher. Altogether, three groups of children were concomitantly performing the obstacle course and three groups the balance beam test. Both motor skill tests were performed individually. Afterwards, the shuttle run test was performed in two consecutive groups consisting each of 6-11 children. All outcomes were measured by specially trained researchers.

#### Shuttle run test

Aerobic fitness was assessed by the multistage 20 m shuttle run test [24]. The test measures aerobic capacity by running back and forth for 20 m with an initial running speed of 8.0 km/h and a progressive 0.5 km/h increase in the running speed every minute indicated by a sound. The maximal performance was determined when the child could no longer follow the pace or stopped because of exhaustion. The test results were expressed as stages (one stage corresponds approximately to the running time of one minute). The 20 m shuttle run test has been found to be a reliable (test-retest *r *= 0.73-0.93 (*p *< 0.05) in 6-to 16-year-old children) [24] and valid measure of maximum oxygen consumption as measured by treadmill testing (*r *= 0.69-0.87, *p *< 0.05) [25]. Due to the young age of the children, some formal adaptations of the original test were made by marking tracks on the floor for each individual child and by having an adult running with the children until the end of the test to provide adequate pace. In one of our pilot studies testing these adaptations, scores were measured twice for children aged 4- to 6 years (*n = *20) and test-retest reliability was *r = *0.84 (*p *< 0.001).

#### Obstacle course

Agility was assessed by an obstacle course that measured a combination of speed, strength, spatial orientation and memorization of a specific sequence of actions [26]. This obstacle course was designed for 3- to 6-year-old children by Vogt and modified by Kunz [27]. It includes running 1 m from a marking cone to a transversally positioned bench, jumping over the bench (36 cm high, 28 cm wide), crawling back under this bench and running back to the marking cone three times in a row as fast as possible. The test was assessed by the time needed to complete the obstacle course and was measured in seconds. Each child had two attempts and the faster one was used for data analysis. The interobserver correlation and the test-retest reliability in our pilot study (*n *= 14) were *r = *0.99 (*p *< 0.01) and *r *= 0.82 (*p *< 0.01), respectively.

#### Balance beam

Dynamic balance was tested by balancing forward barefoot on a 3 m long and 3 cm wide balance beam [28]. As an outcome measure, the consecutive successful steps on the beam were counted until the child's foot touched the floor or until the maximum of eight successful steps was reached. Children performed three trials and the mean of the best two trials was calculated and used for further analyses. The interobserver correlation and the test-retest reliability between the two better attempts in our pilot study (*n *= 15) were *r *= 0.97 (*p *< 0.01) and *r *= 0.84 (*p *< 0.01), respectively.

### Spatial working memory and attention

The tests of spatial working memory and attention were assessed in the preschool setting along with the anthropometric measurements. For the cognitive parameters, children were tested individually in a separate room close to their classroom. All outcomes were measured by specially trained researchers.

#### IDS

To measure spatial working memory performance, a validated subtest for preschoolers that has been taken from the Intelligence and Development Scales (IDS) [29] was applied. Thereby geometrical forms had to be memorized and recognized from a new set of forms including color as distractor. In the first step, one colored geometrical form on a picture was shown that had to be memorized and recognized on a following picture where altogether three forms were shown. Thereby the color of the form changed between the two pictures. Over the following steps the number of forms that had to be memorized within one set of forms rose continuously up to the children's limit. The child had to point at the correct form(s). For every correct set of forms, children received one point. The test was stopped after three consecutive wrong answers. The sum of points was used for further analysis. Significant correlations to related measures confirmed construct validity (i.e. HAWIK-IV Working memory scale: *r *= 0.52) and the test-retest reliability was *r *= 0.48 [30].

#### KHV-VK

To measure attention performance, a validated test for preschoolers („Konzentrations-Handlungsverfahren für Vorschulkinder (KHV-VK)") was applied [31]. The test material consisted of 44 cards with familiar pictures, which had to be sorted into four different boxes. On each card, 12 different small items were visible. Thereby, children had to recognize if among these 12 items, they saw a tree, a hair comb, both or neither of both items. The cards had to be sorted as fast as possible in the four respective boxes showing the same pictures of these items: "a picture of a tree", "a picture of a hair comb", "a picture of both" and "a picture without any items". Total attention score was based on sorting time and error quote. Published test-retest reliability was *r *= 0.88 [31].

### Covariates

*BMI*. Body height and weight were measured using standardized procedures [23]. BMI was calculated as weight (kg)/height (m)^2^.

#### Sociocultural characteristics

To assess migrant status and parental education, parents filled in a general questionnaire regarding their sociocultural characteristics. Parents were defined as migrant if they were born outside of Switzerland [32]. Their educational level was recorded as the respective highest grade of school completed (5 levels). Low educational level was defined as no education beyond mandatory school (9 years) [33]. For analyses, migrant status and low parental education were divided into two categories (at least one vs. no migrant parent/at least one vs. no parent with low education). Children were also categorized into two groups according to the language most frequently spoken at home (native language): French/German (local official languages) vs. other native languages. Due to school legislation, no information could be obtained on economic status.

### Statistics

All analyses were performed using STATA version 11.0 (Statacorp, College Station, Tx, USA). Differences in age, sex and outcome parameters between children with and without complete data were assessed using the unpaired t-test for continuous and the χ^2^-test for binomial variables. All testing was two-tailed and at a significance level of 0.05. For **descriptive analyses**, measures of BMI, sociocultural characteristics, aerobic fitness, motor skills, spatial working memory and attention are presented as means (standard deviations, SD). To assess the relationships between aerobic fitness, motor skills, cognitive parameters and the measured **confounder variables **that are hypothesized to influence aerobic fitness, motor skills and/or cognitive parameters (e.g. age, sex, BMI, sociocultural characteristics like migration status, parental education, native language and linguistic region), we used correlation analyses for continuous confounder variables and t-tests for binomial variables. To assess longitudinal changes of all measures, **mixed linear regression models **were computed. To account for potential clustering of data within preschool classes, all performed regression analyses included respective random intercepts. To assess the cross-sectional and longitudinal relationship of aerobic fitness and motor skills with memory and attention, we used **mixed linear regression models **with the cognitive parameters as outcome and aerobic fitness/motor skills (predictor) as fixed factor. In the longitudinal analysis, we additionally adjusted for baseline memory and attention to assess the association between baseline aerobic fitness/motor skills and the prospective change in cognitive parameters. In a second step ("**adjusted model**"), we adjusted cross-sectional and longitudinal analyses for the potential confounder variables. For better understandability and comparability, the results of mixed linear models were also expressed in the form of **partial correlation coefficients**. These coefficients were computed by first regressing outcome and predictor variables of interest against the covariates of the underlying model and then correlating the resulting residuals. Conversely, the same regression and correlation analyses were performed with aerobic fitness/motor skills as the outcome and cognitive parameters as predictor variables. **Potential interactions **with sex were tested. As no sex interactions were found, we did not stratify analyses by sex.

## Results

The **characteristics of the sample **at baseline and at follow up are shown in Table 1. 78% of the children had a migrant background and the most frequent migrant countries or regions were: Portugal, Africa, Asia, Turkey, Albania and "rest of Europe". Aerobic fitness, motor skills, spatial working memory and attention improved over the 9 months. Analyses between the confounder variables and the outcome measures showed that aerobic fitness, motor skills and cognitive parameters were related to age (*p*≤0.002), while only aerobic fitness and motor skills were related to BMI (all *p *< 0.01). Aerobic fitness, motor skills and both cognitive parameters differed according to sociocultural characteristics (*p *< 0.05 for differences according to either migration status, parental education, native language and/or linguistic region). Baseline and follow up values were correlated as following: aerobic fitness (*r *= 0.76), agility (*r *= 0.71), dynamic balance (*r *= 0.33), working memory (*r *= 0.42), attention (*r *= 0.31), all *p *< 0.001.

**Table 1:** Descriptive characteristics of the 245 children (girls *n = *121, boys *n = *124) at baseline and follow up

	Baseline	Follow up	*p*-value
**Age and anthropometry**			
Age [years], mean (SD)	5.2 (0.6)		
BMI [kg/m^2^], mean (SD)	15.8 (1.6)	15.9 (1.7)	0.05

**Sociocultural characteristics**			
Migration background^1^, yes/no [%]	79/21		
Foreign language spoken at home, yes/no [%]	41/59		
Low parental education^2^, yes/no [%]	44/56		

**Physical fitness**			
Agility [s], mean (SD)	19.2 (4.5)	16.6 (3.2)	<0.001
Aerobic fitness [stages], mean (SD)	3.0 (1.4)	4.5 (1.7)	<0.001
Dynamic balance [steps], mean (SD)	2.4 (1.7)	3.0 (2.0)	<0.001

**Cognition**			
Spatial working memory [no. of points], mean (SD)	3.6 (2.0)	4.8 (1.7)	<0.001
Attention score [standard nine values], mean (SD)	10.4 (2.3)	11.0 (2.1)	<0.01

Mixed linear regression analyses with preschool class (cluster) as random factor. SD, standard deviation; s, seconds.

^1 ^at least 1 parent born outside Switzerland vs. no parent born outside Switzerland

^2 ^at least 1 parent with no education beyond mandatory school (9 year) vs. middle or high parental education

In the **cross-sectional analyses **(Table 2), **aerobic fitness **was positively related to attention in the adjusted analyses. The relationship of **aerobic fitness **with working memory, however, did not remain significant after adjustment. A shorter time in the obstacle course (increased level of **agility**) was related to better performance in working memory and in attention before and after adjustment. The relationship of **dynamic balance **with working memory did not remain significant after adjustment.

**Table 2:** Cross-sectional relationship between baseline aerobic fitness and motor skills with cognition (*n = *245).

	Aerobic fitness [stages]			
	
	**β-Coeff**.	95% CI	*p*-value	r^3^
	
Spatial working memory [no. of points]				
Unadjusted model^1^	0.30	0.12 to 0.47	0.001	0.22
Adjusted model^2^	0.12	-0.08 to 0.33	0.246	0.08
Attention score [standard nine values]				
Unadjusted model^1^	0.19	-0.09 to 0.47	0.850	0.10
Adjusted model^2^	0.25	0.02 to 0.49	0.036	0.15
	**Agility [s]**			
	
	**β-Coeff**.	**95% CI**	***p*-value**	**r^3^**
	
Spatial working memory [no. of points]				
Unadjusted model^1^	-0.12	-0.17 to -0.06	**<0.001**	-0.27
Adjusted model^2^	-0.07	-0.13 to -0.02	**0.013**	-0.17
Attention score [standard nine values]				
Unadjusted model^1^	-0.10	-0.16 to -0.02	**0.008**	-0.18
Adjusted model^2^	-0.11	-0.19 to -0.03	**0.005**	-0.20

	**Dynamic balance [steps]**			
	
	**β-Coeff**.	**95% CI**	***p*-value**	**r^3^**
	
Spatial working memory [no. of points]				
Unadjusted model^1^	0.17	0.02 to 0.3	**0.024**	0.14
Adjusted model^2^	0.02	-0.13 to 0.17	0.792	0.02
Attention score [standard nine values]				
Unadjusted model^1^	0.27	0.01 to 0.53	0.774	0.13
Adjusted model^2^	0.06	-0.11 to 0.24	0.452	0.06

Mixed linear regression analyses with preschool class (cluster) as random factor.

95%CI, 95% confidence interval; β-Coeff., β-Coefficient; s, seconds

^1 ^not adjusted

^2 ^adjusted for sex, age, BMI, migration status, parental education, native language and linguistic region

^3 ^partial correlation coefficient between outcome and predictor variable.

In the **longitudinal analyses **(Table 3), baseline aerobic fitness was associated with improvements in attention. The relationship of an increased level of baseline agility with improvements in memory and in attention over nine months did not remain significant after adjust-ment. However, baseline dynamic balance was associated with improvements in spatial working memory before and after adjustment.

**Table 3:** Longitudinal relationship between baseline aerobic fitness and motor skills with changes in cognition 9 month later (*n = *245).

	Aerobic fitness [stages]			
	
	**β-Coeff**.	95% CI	*p*-value	r^3^
	
Spatial working memory [no. of points]				
Unadjusted model^1^	0.1	-0.03 to 0.24	0.136	0.1
Adjusted model^2^	0.08	-0.09 to 0.24	0.366	0.06
Attention score [standard nine values]				
Unadjusted model^1^	0.29	0.11 to 0.47	0.002	0.19
Adjusted model^2^	0.25	0.03 to 0.48	0.029	0.16
	**Agility [s]**			
	
	**β-Coeff**.	**95% CI**	***p*-value**	**r^3^**
	
Spatial working memory [no. of points]				
Unadjusted model^1^	-0.05	-0.09 to -0.01	**0.020**	-0.15
Adjusted model^2^	-0.04	-0.09 to 0.01	0.100	-0.11
Attention score [standard nine values]				
Unadjusted model^1^	-0.07	-0.14 to -0.005	**0.036**	-0.14
Adjusted model^2^	-0.05	-0.12 to 0.03	0.241	-0.09

	**Dynamic balance [steps]**			
	
	**β-Coeff**.	**95% CI**	***p*-value**	**r^3^**
	
Spatial working memory [no. of points]				
Unadjusted model^1^	0.17	0.06 to 0.28	**0.002**	0.20
Adjusted model^2^	0.13	0.01 to 0.25	**0.035**	0.15
Attention score [standard nine values]				
Unadjusted model^1^	0.13	-0.02 to 0.3	0.081	0.12
Adjusted model^2^	0.05	-0.11 to 0.22	0.518	0.05

Mixed linear regression analyses with preschool class (cluster) as random factor.

95%CI, 95% confidence interval; β-Coeff., β-Coefficient; s, seconds

^1 ^adjusted for baseline cognitive performance

^2 ^adjusted for sex, age, BMI, migration status, parental education, native language, linguistic region and baseline cognitive performance

^3 ^partial correlation coefficient between outcome and predictor variable.

In case of a loss of significance, this was not due to the lower sample size in the adjusted model. The relationships of aerobic fitness and motor skills with working memory were mostly attenuated by the age of the children, and the ones with attention by parental education and by BMI. In contrast to the above mentioned relationships, baseline memory and attention were not associated with any improvements in aerobic fitness or motor skills, neither before nor after adjustment (all *p *> 0.1).

## Discussion

### Main results

These analyses addressed both the cross-sectional and longitudinal relationships of aerobic fitness and motor skills with spatial working memory and attention in preschool children. Aerobic fitness and motor skill measures were positively associated with current working memory and/or attention after adjustment for BMI and sociocultural characteristics, and to some extent also with their future improvements over the following 9 months. Thereby, the relationship varied if aerobic fitness, agility or balance were investigated: The cross-sectional results were most consistent for agility where both physical and cognitive resources are re-quired.

### Relation of baseline aerobic fitness and motor skills with baseline memory and attention

In our cohort, aerobic fitness and motor skills were positively related to current cognitive parameters. The findings concerning the motor skill measures complement two existing cross-sectional studies in preschoolers that show similar relationships [13,14]. One previous study that failed to show a relationship between motor skills and cognition had a small sample size (*n = *36 children) [17] compared to the other two (*n = *85 and 295) [13,14]. However, none of the preschool studies assessed aerobic fitness and they did not adjust for BMI or sociocultural characteristics. As our data demonstrate, it is important to account for these confounders.

### Relation of aerobic fitness and motor skills with improvements in memory and attention over 9 month

Our longitudinal analysis showed that baseline aerobic fitness and motor skills were related to some improvements in cognitive parameters. However, the relationships were not always consistent with the cross-sectional results and were sometimes attenuated after controlling for confounders. One possible explanation is that the positive relation of aerobic fitness or motor skills with memory or attention might be set earlier for certain parameters with no further amplification of differences during the preschool years. It might be, that there are sensitive periods, during which aerobic fitness and motor skills have a stronger impact on cognitive parameters. And possibly the time period of nine month is too short to detect a relation between two measures, which are both strongly associated with other factors. One previous longitudinal study has been performed in young children that assessed the relationship of reported motor performance between birth and age 4 to cognitive parameters at age 6 [21]. This study found that motor performance accounted for a significant proportion of the variance of cognitive parameters. However, they did not control for baseline cognitive parameters and thus could not assess longitudinal changes. Additionally, motor performance was only reported, but not measured.

### Relations according to aerobic fitness and motor skills

Concerning **aerobic fitness**, the present study has found a cross-sectional relationship of aerobic fitness with attention in the adjusted analyses. In the longitudinal analyses, aerobic fitness was consistently related to improvements in attention. This would indicate that high fitness in preschool children might have beneficial effects on attention in the following years. These longitudinal data are in line with a cross-sectional study done by Hillman and his colleagues, which revealed that aerobic fitness in school children was associated with accuracy and speed (both measured in our attention task) in a stimulus discrimination task [12]. However, in contrast to the predominantly adult literature, we did not find a consistent relationship between aerobic fitness and memory. In accordance with previous reviews [18,19], we conclude that factors that influence cognitive development in children might probably differ from those in adults and that the relationship of aerobic fitness with cognitive performance might evolve over time.

Of the domains assessed in the present study, **agility **was most consistently related to both cognitive measures in the cross-sectional analyses. Agility was tested through an obstacle course that measured a combination of speed, strength, spatial orientation and memorization of a specific sequence of actions. This kind of resource-demanding test involves both physical and cognitive resources [26] which could explain the correlation with both cognitive measures. Similar results have been found in a study with sport games which are also considered to require both physical and cognitive resources [34].

We found a relationship of **dynamic balance **with working memory that got more consistent when analyzed longitudinally, possibly due to maturation and neural adaptations that occur in young children. No association was found between dynamic balance and attention. These results coincide with cross-sectional findings in preschoolers with and without learning disabilities, where the authors did not find a positive relation of dynamic and static balance with global aspects of cognition, but with working memory [35]. Both the balance and the memory task do not include speed in their evaluation which might be one reason for the found relationship.

### Confounder variables

In our population, some associations were attenuated or did not remain significant after ad-justment for different confounder variables. In another longitudinal study [21] and in a cross-sectional study in older children [36] the adjustment for sociocultural characteristics attenuated [36] or strengthened [21] the relationships between physical fitness/motor skills and academic achievement or cognitive parameters. This underlines the importance to take those confounders into account.

### Relation of baseline memory and attention with improvements in aerobic fitness or motor skills

In contrast to the relationships mentioned above, we did not find any associations of baseline memory and attention with improvements in aerobic fitness and motor skills, neither before nor after adjustment. This lack of longitudinal relationship has not been previously investigated and adds to our understanding regarding the dominant direction of the relationship between those measures.

### Strength and Limitations

We included a comprehensive and objective assessment of aerobic fitness, two different mo-tor skill measures and two cognitive parameters in a relatively large sample of preschool children. An important strength of this study is the cross-sectional and longitudinal design in the same population, which is novel for this young age group. This also gives a hint about the direction of the observed relationships despite the relatively short follow-up period. Although we did not directly measure academic achievement, attention and working memory may play a crucial role in key learning areas for children at the beginning of formal education [37]. For example, measures of working memory at school entry have been found to provide excellent predictors of children's success in national assessments of scholastic abilities up to 3 years later [38]. Unfortunately, the test-retest reliability of the spatial working memory task is with r = 0.48 quite low. Another limitation is the use of an indirect measurement of VO_2 _to test aerobic fitness. This may have diluted the relationship between aerobic fitness and cognitive parameters. However, the test had a good reproducibility in our pilot and laboratory tests would not have been feasible in this epidemiological approach. In this age group, it can be hypothesized that tests conducted in the preschool setting may better reflect performance levels in real-life than assessments in more experimental settings [39].

## Conclusions

Based on our results, higher performance in aerobic fitness and motor skills in preschoolers seems to be related to improved spatial working memory and/or attention. Based on neurophysiological approaches, we cautiously suggest that structural and functional modifications might explain the relationship of aerobic fitness and motor skills with cognitive parameters found in our study. Baseline measurements were to some extent also related with improvements in memory and attention over nine month. Based on our results we suggest that exercises involving specific mental processing including executive functions are most suitable to trigger global cognitive development in young children. Further studies should measure the longer-term impact of increasing aerobic fitness and/or motor skills as well as the impact of different domains on specific cognitive parameters. Our data contribute to the emerging field of brain fitness and highlight the importance of a promotion of physical education.

## Competing interests

The authors declare that they have no competing interests.

## Authors' contributions

IN was implicated in the more detailed conception of the study, responsible for the perfor-mance of the aerobic fitness and motor skills tests in half of the children, had a main job in the data collection, analyzed and presented all data, wrote the initial manuscript and all its revisions. SK was of major assistance in the conception of the project, the choice of the aerobic fitness and motor skills tests and the physical activity measurements as well as the presentation of the data. JG supported us in the analysis and the interpretation of the results as well as in revising the manuscript giving inputs of a psychologist. One of the cognitive tests used (IDS), was provided to us from her research group. TH helped in the choice and performance of the cognitive tests, contributed in the data collection and assisted in presentation of the data. JB supported the team in the neurocognitive aspects, in the data collection and in the presentation of the data. CS assisted in the conception of the study and gave epidemiological and statistical assistance. JP is the project leader for the design, implementation, analysis and presentation of the data, the draft and all revisions of the manuscript. All authors read and approved the final manuscript.

## Pre-publication history

The pre-publication history for this paper can be accessed here:

http://www.biomedcentral.com/1471-2431/11/34/prepub
